# Finasteride inhibits melanogenesis through regulation of the adenylate cyclase in melanocytes and melanoma cells

**DOI:** 10.1007/s12272-018-1002-x

**Published:** 2018-02-03

**Authors:** Jae Ok Seo, Silvia Yumnam, Kwang Won Jeong, Sun Yeou Kim

**Affiliations:** 1 College of Pharmacy, Gachon University, #191, Hambakmoero, Yeonsu-gu, Incheon, 406-799 Republic of Korea; 2 Gachon Medical Research Institute, Gil Medical Center, Incheon, Republic of Korea; 3 Gachon Institute of Pharmaceutical Science, Gachon University, Yeonsu-gu, Incheon, 409-799 Republic of Korea

**Keywords:** Finasteride, Melan-a, B16F10, MC1R, Melanogenesis

## Abstract

Finasteride is a well-known 5α-reductase inhibitor used for treatment of alopecia and prostate cancer. But the effect of finasteride in regulating melanogenesis is still unclear. In the present study the role of finasteride on melanogenesis was investigated. Finasteride decrease melanin level in melanocyte melan-a cells and B16F10 melanoma cells without inducing cytotoxicity. MC1R (melanocortin 1 receptor) protein expression was also inhibited by finasteride thereby decreasing the expression of adenylate cyclase, MITF (Melanogenesis associated transcription factor), tyrosinases, TRP (tyrosinase-related protein) -1 and -2. Thus our study suggest that finasteride inhibits melanogenesis in melanocyte and melanoma cells by inhibiting MC1R.

## Introduction

Melanocytes are specialized skin cells that determine hair and skin color through the production of pigments called melanin. Additionally, melanocytes shield the body from detrimental environmental factors, such as ultraviolet (UV) light, which can cause melanoma and other skin tumors (Ha et al. 2009; Chou et al. 2013; Lee et al. 2014). Melanocytes are present in the basal lamina and/or the bottom of the epidermis and contain a specialized organelle called melanosome (Choi et al. 2014). A melanosome is a granule containing melanin, which is transferred to neighboring keratinocytes in the epidermis (Lin and Fisher 2007; Lee et al. 2014).

Hormones can regulate melanogenesis in melanocytes. For example, α-melanocyte-stimulating hormone (α-MSH), derived from pro-opiomelanocortin (POMC), significantly enhances the activity of tyrosinase, the main enzyme regulating melanin synthesis. α-MSH activates the production of melanin by binding to the melanocortin1receptor (MC1R) on the cell membrane (Robbins et al. 1993). MC1R is primarily involved in the regulation of skin and hair color and helps prevent UV-induced damage. Additionally, MC1R is a potential target for the stimulation of eumelanin synthesis in melanocytes (Mitra et al. 2012). The binding of α-MSH to MC1R also has anti-inflammatory effects (Kadekaro et al. 2012). Activation of MC1R induces adenylate cyclase and increases cAMP levels, thereby enhancing the expression of MITF (Melanogenesis associated transcription factor), a transcription factor that regulates protein kinase A (PKA) expression. Further, MITF enhances melanogenesis by stimulating the transcription of tyrosinase and tyrosinase-related protein (TRP)-1 and -2 (Dessinioti et al. 2011; Wolnicka-Glubisz et al. 2014).

Sex hormones, particularly estrogen, are known to regulate melanogenesis. Estrogen increases melanin content and melanocytes in human skin, and excessive amounts of estrogen may induce melanoma (Bolognia et al. 1989; McLeod et al. 1994). In a recent report, androgen receptors were present in the skin, and were related to skin melanogenesis (Slominski et al. 2004). Further, testosterone, which is the predominant androgen along with dihydrotestosterone (DHT) in men, influenced melanin synthesis by regulating melanogenic enzymes (Bischitz and Snell 1959). In humans, there are two types of 5α-reductase, Type I and II. Type I 5α-reductase is primarily expressed in the sebaceous gland, whereas Type II 5α-reductase mainly regulates DHT in the prostate, hair follicles, and epididymis (Finn et al. 2006). Finasteride has been used to treat baldness, and has been approved for medical use when down-regulation of Type II 5α-reductase and DHT activity levels is necessary (Rousso and Kim 2014). In this manner, the conversion of testosterone to DHT, progesterone to dihydroprogesterone (DHP), and deoxycorticosterone to dihydrodeoxycorticosterone (DHDOC) can be blocked (Gupta and Charrette 2014; Zabkowski 2014).

Novel therapeutics are necessary for the treatment of androgenic alopecia in men, because its incidence is increasing. Finasteride has been proposed as a treatment for this disorder (Geller and Sionit 1992; Gormley et al. 1992; Vis and Schroder 2009). Recently, (Weinstein et al. 2013) reported that men with red hair are at lower risk for prostate cancer than men with other hair colors, possibly due to polymorphisms in the MC1R gene. This has interesting implications because finasteride is well known for treating prostate cancer and promoting hair growth in men. However, the effect of finasteride on melanogenesis in melanocytes remains unknown. The aim of our work was to examine the impact of finasteride on melanogenesis in vitro using cultured melan-a melanocytes, B16F10 melanoma cells, and normal human epidermal melanocytes (NHEM). In addition, we evaluated the effects of finasteride on melanogenesis.

## Materials and methods

### Materials

Dulbecco’s modified Eagle’s medium (DMEM), fetal bovine serum (FBS), and penicillin–streptomycin (PS) were purchased from Hyclone (Carlsbad, CA, USA), and RPMI1640 was purchased from Gibco-BRL (Gaithersburg, MD, USA). Melanocyte growth medium with supplements was purchased from Promo Cell (Heidelberg, Germany). 12-O-tetradecanoylphorbol-13-acetate (TPA), 1-phenyl-2-thiourea (PTU), kojic acid, Triton X-100, phenylmethylsulfonyl fluoride (PMSF), aprotinin, mushroom tyrosinase, 3,4-dihydroxy-l-phenylalanine (l-DOPA), α-MSH, DMSO, and finasteride were purchased from Sigma-Aldrich (St.Louis, MO, USA). β-defensin-3 was purchased from Prospec (East Brunswick, NJ, USA).

### Cell culture

The murine melanocyte cell line melan-a was supplied by Dr. Byeong Gon Lee from the Skin Research Institute, Amore Pacific Co. (Yongin, Korea). The mouse melanoma cell line B16F10 was obtained from the Korean Cell Line Bank (Seoul, Korea). Primary normal human epidermal melanocytes (NHEM) were purchased from PromoCell (Heidelberg, Germany). RPMI 1640 supplemented with 10% FBS, 1% PS, and 400 nM TPA was used to maintain the melan-a cells. B16F10 cells were maintained in DMEM medium with 10% FBS, and 1% PS, and NHEM cells were in melanocyte growth medium with supplements. Cells are incubated at 37 °C in a humidified incubator with 5% CO_2_.

### Measurement of melanin content

Melan-a cells were seeded in a 24-well plate (1 × 10^5^ cells/well) and were treated with various concentration of finasteride for 72 h. Melanin content was measured after 72 h, using a modification of the method as described by (Hosoi et al. 1985). Briefly, after removing the media, cells were washed twice with phosphate-buffered saline (PBS). Sodium hydroxide solution (1 ml, 1 N) was added to each well to dissolve the melanin. Melanin absorbance was measured at 405 nm using a microplate reader. This assay was repeated with B16F10 cells (2 × 10^5^ cells/well) using the same method.

### Measurement of cell viability

Cell viability was determined using a 3-[5-dimethylthiazol-2-yl]-2,5-diphenyltetrazolium bromide (MTT) assay. B16F10 and melan-a cells were seeded in a 24-well plate (1 × 10^5^ cells/well) and treated with 0, 1, 10, 20 or 100 µM and 0, 0.1, 1 and 10 µM finasteride respectively, which was supplemented every 24 h, for 72 h. The media were removed and cells were washed with PBS. Thereafter, 400 µl of 0.1% MTT reagent was added and the plates were incubated for 1 h. MTT reagent was then removed and DMSO was added. Plates were placed on a shaker for 10 min, and the absorbance was measured at 570 nm using a microplate reader. The same method was used to evaluate B16F10 cell viability.

### Mushroom tyrosinase activity

To estimate the inhibitory effects of finasteride on mushroom tyrosinase activity, tyrosinase was incubated with 0.1, 1 or 10 µM finasteride or kojic acid, as a positive control. Each sample was dissolved in methanol. l-DOPA (8.3 mM) and mushroom tyrosinase (125 U) were diluted in 80 mM phosphate buffer (pH 6.8). First, 40 µl of each sample and 120 µl of l-DOPA were mixed in a 96-well plate, then 40 µl of diluted mushroom tyrosinase was added. The plates were incubated for 15 min and absorbance was measured at 490 nm using a microplate reader.

### Tyrosinase activity in melan-a cells

Murine melan-a cells were seeded in 100 mm dishes (1 × 10^6^ cells/dish) and incubated for 3 days. After washing with PBS, all cells were incubated with 600 µl of trypsin–EDTA for 3 min at 37 °C. Detached cells were suspended in 1 ml PBS, and transferred to a centrifuge tube. Cells were then centrifuged at 7500×*g* for 5 min, and PBS was removed. Tyrosinase buffer (80 mM phosphate buffer, 1% Triton-X 100, 100 µg/ml PMSF) was added to the cell pellets, and the suspension was ultra-sonicated on ice. After centrifugation at 12,500×*g* for 20 min at 4 °C, the supernatant was used for the tyrosinase assay. Protein content was measured using bovine serum albumin (BSA) as a standard. For each reaction, 150 µg of protein was used.

Tyrosinase activity was measured by determining the rate of l-DOPA oxidation, as reported by Shono et al. To estimate the inhibitory effects of finasteride on melan-a cell tyrosinase, 40 µl of finasteride in methanol (0.1, 1 or 10 µM), or the positive control kojic acid, was added to a 96-well plate with 120 µl of l-DOPA and 150 µg of protein. After mixing, the plates were incubated for 15 min, and the absorbance was measured at 490 nm using a microplate reader.

### In situ l-DOPA staining in cells

B16F10 and melan-a cells were seeded in a 24-well plate and incubated for 72 h with finasteride. Cells were fixed with 4% paraformaldehyde for 40 min, followed by treatment with 0.1% triton X-100 for 2 min. l-DOPA (0.1%) was added to each well and the plates were incubated for 3 h. The cells were washed twice with PBS and observed under a microscope.

### Western blot analysis

Melan-a cells were seeded in 100 mm dishes (1 × 10^6^ cells/dish) and treated with 0.1, 1, or 10 µM finasteride for 3 days at 37 °C. Cells were then washed with PBS and harvested with trypsin–EDTA. Detached cells were gathered in 1 ml of PBS and centrifuged at 7500 rpm for 5 min. Cell pellets were lysed using lysis buffer (50 mM Tris–HCl, pH 8.0, 0.1% SDS, 150 mM NaCl, 1% NP-40, 0.02% sodium azide, 0.5% sodium deoxycholate, 100 µg/ml PMSF, 1 µg/ml aprotinin) for 1 h on ice. The lysates were centrifuged at 12,500 rpm for 20 min at 4 °C, and the supernatant was used for western blotting. The protein content was measured using BSA as a standard. Protein (40 µg) was separated using a 12% SDS-PAGE gel and transferred to nitrocellulose membranes. The membranes were blocked with 5% skim milk for 1 h, and incubated overnight with primary antibodies targeting α-tubulin (1:3000, Sigma), MITF (1:500, Cell Signaling), tyrosinase (1:500, Cell Signaling), 5-α-reductase (1:200, Santa Cruz), MC1R (1:200, Santa Cruz), TRP-1 (1:500, Santa Cruz), TRP-2 (1:500, Santa Cruz) or adenylate cyclase (1:500, Santa Cruz) at 4 °C. After removing the primary antibodies, membranes were washed three times with TBST and incubated with secondary antibodies (goat anti-mouse IgG: Thermo scientific, donkey anti-goat IgG-HRP, goat anti-rabbit HRP: Santa Cruz) for 1 h. The membranes were treated with enhanced chemi-luminescence reagent using ChemiDocXRS + imaging system (Bio-Rad, California, USA).

### Statistical analysis

The data were analyzed using Statistical Analysis System (SAS) software. All data are expressed as the mean ± SEM. Statistical comparisons between different treatments were performed using one-way ANOVA with Turkey’s multiple comparison post-test and *p* values less than 0.05 were considered statistically significant.

## Results

### Finasteride decreased the melanin content in melanocyte and melanoma cell lines

**Fig. 1:**
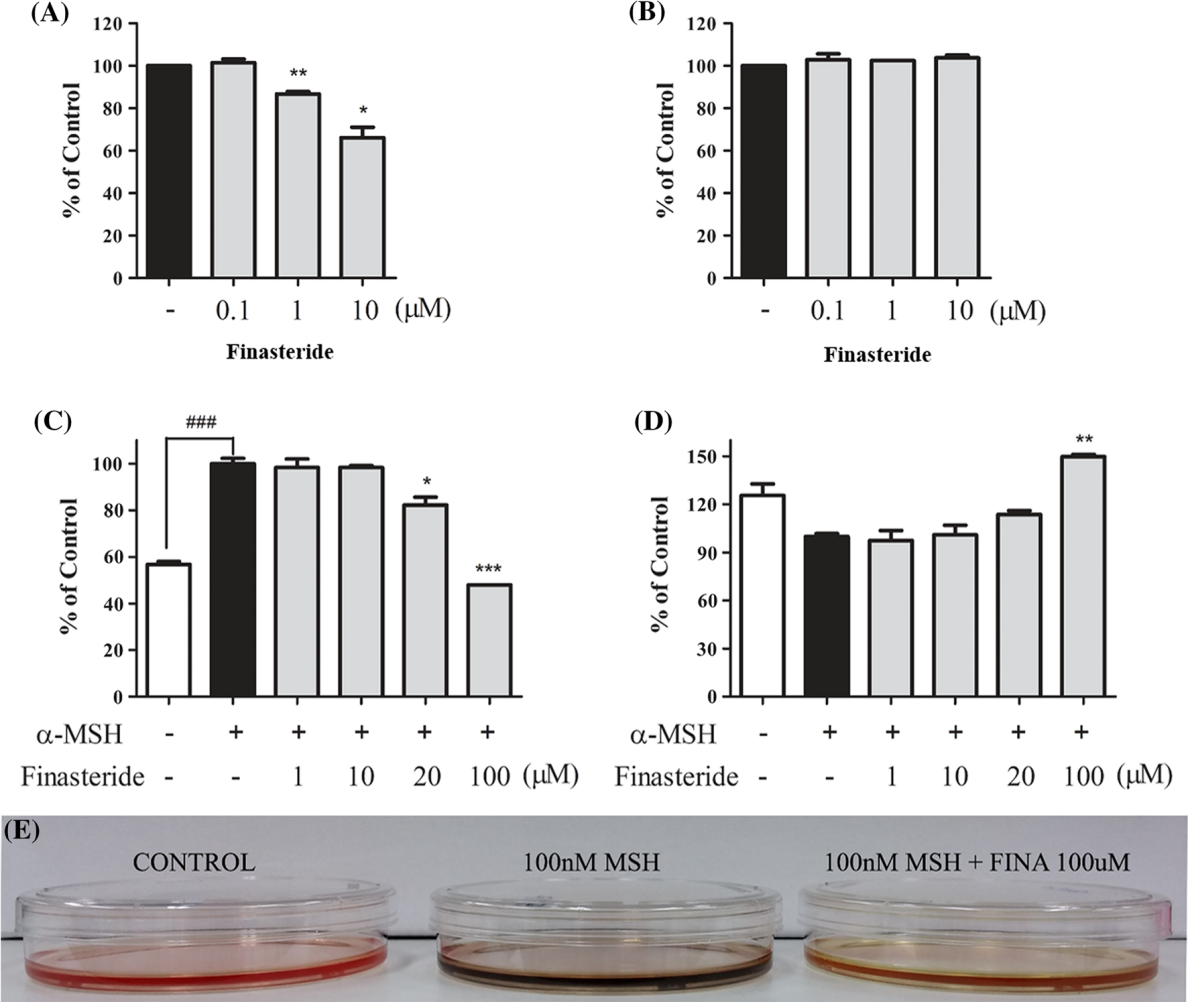
Inhibitory effects of finasteride on melanin contents and cell viability in Melan-a and B16F10 cells. Cells were treated with the indicated concentration of finasteride for 72 h. **a** Melanin content and **b** cell growth rate were measured in melan-a cells. **c** Amount of melanin and **d** cell growth rate in B16F10 cells with µ nM of α-MSH. White bar represent untreated cells and black bars represent α-MSH-treated cells. All data are expressed as mean ± SEM, and were analyzed by one-way ANOVA, followed by the Student’s *t* test. *p < 0.05 indicates that the treatment group is significantly different from the α-MSH-treated control group (*p < 0.05, **p < 0.01 and ***p < 0.001) and ###p < 0.001 indicate a significant difference versus the untreated group. **e** B16F10 cells were treated with α-MSH and 100 µM of finasteride

To evaluate the effects of finasteride on melanin content and cytotoxicity, melan-a cells were treated with increasing concentrations of finasteride (0, 0.1, 1 and 10 µM). The melanin content decreased to 66% following treatment with 10 µM finasteride (Fig. 1a). Interestingly, 10 µM finasteride did not have any effect on melan-a cell viability, indicating that finasteride was non-toxic to melan-a cells and may decrease melanogenesis (Fig. 1b).

Melanin content and cell viability of B16F10 cells were also measured using MTT assay after treatment with increasing doses of finasteride (0, 1, 10, 20 or 100 µM) with α-MSH for 3 days (Yang et al. 2011). Interestingly, treatment with 100 µM finasteride significantly decreased melanin content which was induced by α-MSH, without any cell death (Fig. 1c, d). These results suggested that finasteride reduced melanogenesis both in melan-a and B16F10 cells.

### Finasteride inhibits in situ tyrosinase activity

**Fig. 2:**
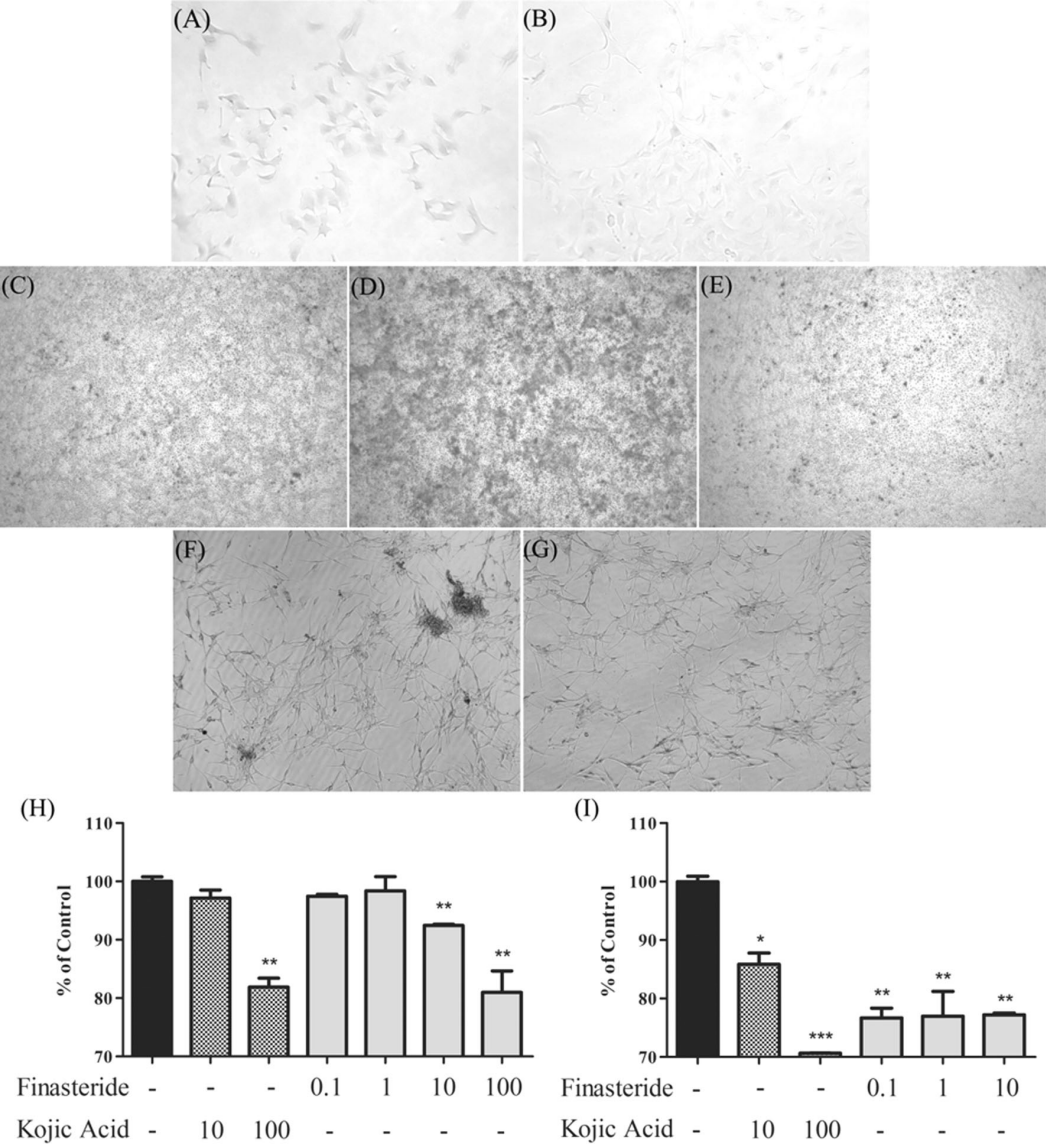
Inhibitory effects of finasteride on DOPA and tyrosinase activity. **a** Melan-a cells were incubated in the absence or **b** presence of 10 µM finasteride. **c** B16F10 cells were incubated without treatment, **d** with 100 nM α-MSH, or with **e** 100 µM finasteride and 100 nM α-MSH. **f** Untreated NHEM cells and **g** cells treated with 10 µM finasteride. **h** Inhibitory effects of finasteride on mushroom tyrosinase activity. **i** Inhibitory effects of finasteride on tyrosinase activity in melan-a cells. Cells were treated with finasteride (0.1, 1, or 10 µM) and the amount of DOPA-chrome was measured. Kojic acid was used as a positive control. All data are expressed as the mean ± SEM, and were analyzed by one-way ANOVA, followed by the Student’s *t* test. *p < 0.05 indicates that the treatment group is significantly different from the control group (*p < 0.05 and **p < 0.01)

Tyrosinase is the rate-limiting enzyme that regulates melanogenesis (Slominski et al. 2012). To establish the effect of finasteride on tyrosinase activity in melanocytes and melanoma cells, l-DOPA staining was performed. Staining indicated a clear representation of the synthetic ability of tyrosinase in cells. Cells were incubated with finasteride, then with l-DOPA. Compared to control, melan-a cells treated with 10 µM finasteride exhibited decreased melanin synthesis (Fig. 2a, b). In addition, it was observed that finasteride decreased the α-MSH induced melanin synthesis in B16F10 cells (Fig. 2c, d, e). Furthermore, finasteride decreased the l-DOPA level in NHEM cells compared to that in untreated cells (Fig. 2f, g). These results suggested that finasteride inhibited tyrosinase activity both in melanocytes and melanoma cells.

### Effect of finasteride on tyrosinase activity

Tyrosinase activity plays a key role in melanogenesis. To examine whether finasteride inhibited tyrosinase activity, the amount of DOPA chrome was measured. Kojic acid, a well-known tyrosinase inhibitor was used as a positive control. Treatment with finasteride caused a significant decrease in mushroom tyrosinase activity compared with that in control cell (Fig. 2h). Moreover, finasteride significantly reduced tyrosinase activity in melan-a cells. As shown in Fig. 2i, finasteride treatment decreased tyrosinase activity by 20% compared to that in control cells. Therefore, the results indicate that finasteride inhibits tyrosinase activity in melan-a cells.

### Finasteride inhibited the expression of melanogenic enzymes and decreased MITF expression in melan-a cells

**Fig. 3:**
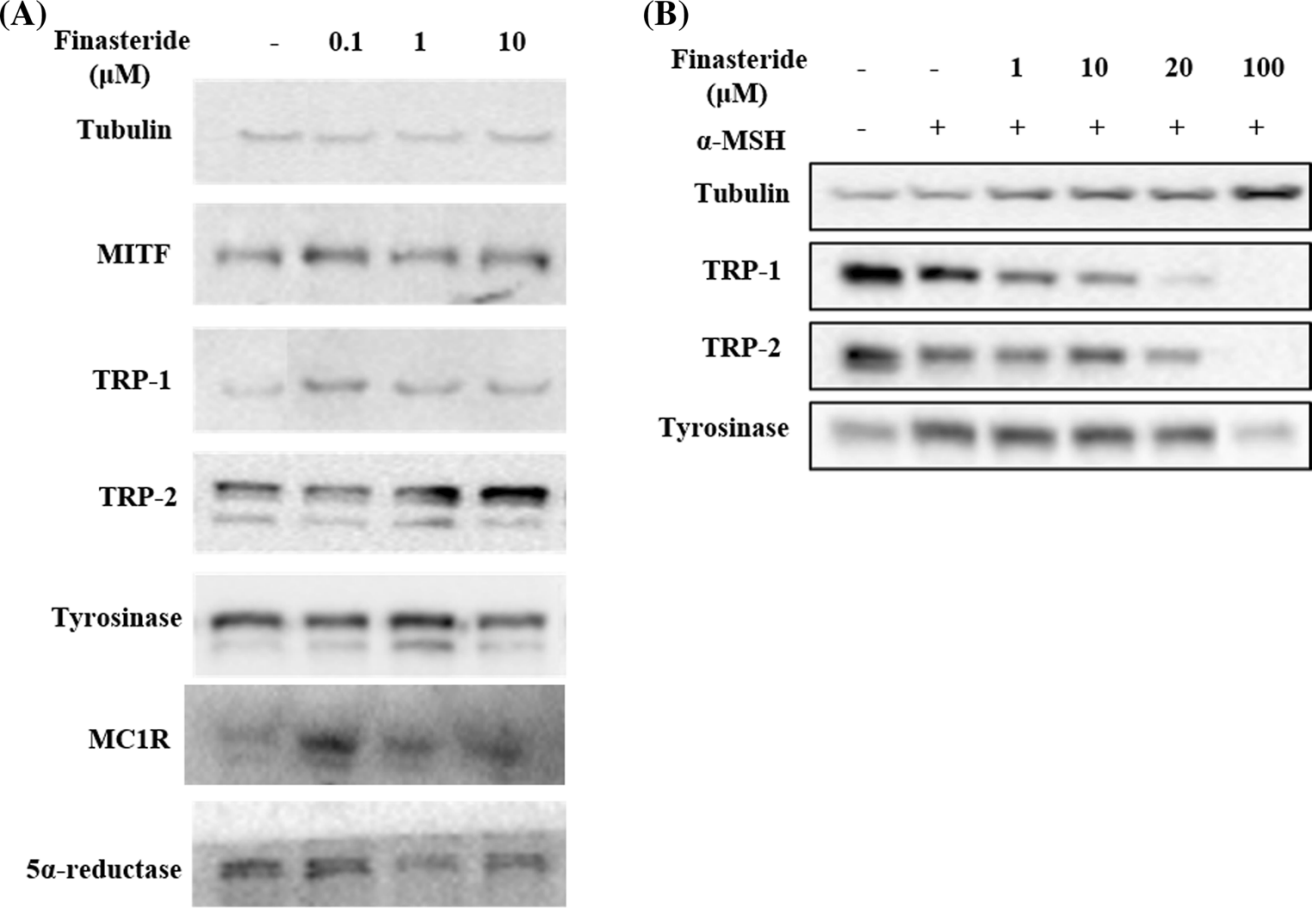
Inhibitory effects of finasteride on melanogenic enzyme expressions. **a** Melan-a cells were treated with finasteride (0.1, 1, or 10 µM) for 72 h. **b** B16F10 cells were co-treated with 100 nM α-MSH and indicated concentration of finasteride. Equal amounts of protein (40 µg/lane) were separated using a 12% SDS–PAGE gel, and were detected using specific antibodies. Equal protein loading was evaluated using antibodies against α-tubulin

To confirm that finasteride-mediated melanin inhibition was correlated with the expression of melanogenic enzymes, such as tyrosinase, TRP-1, TRP-2, and MITF, western blots analysis was performed in melan-a cells. Treatment with finasteride dose-dependently decreased the protein expression of tyrosinase, TRP-1, TRP-2, MITF, MC1R and 5α-reductase. α-MSH increased the protein expression of tyrosinase, TRP-1, TRP-2, MC1R and MITF in B16F10 cells but when cells were co-treated with finasteride the increase in protein expression of tyrosinase, TRP-1, TRP-2, MC1R and MITF were reduced (Fig 3). These results suggest that finasteride inhibit melanogenesis in melan-a and B16F10 cells.

### Effect of finasteride on adenylate cyclase

**Fig. 4:**
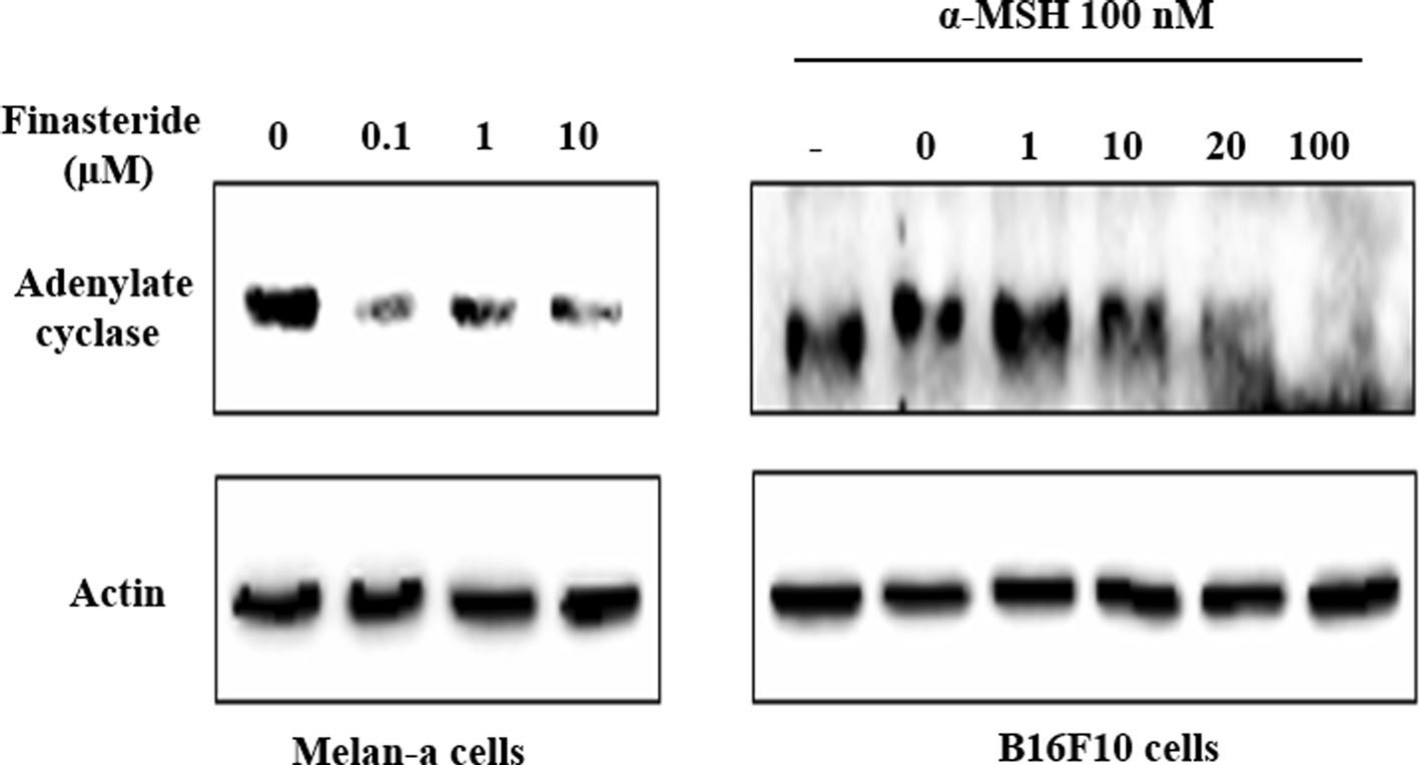
Inhibitory effects of finasteride on adelyate cyclase enzyme: Melan-a cells and B16F10 cells were treated with indicated concentration of finasteride for 72 h. Equal amounts of protein (40 µg/lane) were separated using a 12% SDS–PAGE gel, and were detected using specific antibodies. β-actin was used as an internal control

The binding of α-MSH to MC1R receptors induced intracellular signals, primarily elevating intracellular cAMP by adenylate cyclase (Mountjoy et al. 1992), resulting in the activation of genes involved in melanogenesis. A previous study indicated that the expression of adenylate cyclase was increased by testosterone in rat seminal vesicles (Ramli et al. 2016). Since finasteride treatment resulted in a decrease of MITF expression (Fig. 3) which is known to be driven by adenylate cyclase, we investigated the effect of finasteride on the protein expression of adenylate cyclase in melanocytes. Treatment with finasteride (0.1–10 µM) decreased the protein level of adenylate cyclase in melan-a cells (Fig. 4). A similar reduction of adenylate cyclase was observed in B16F10 cells treated with finasteride (1–100 µM), suggesting that finasteride blocked the activation of MITF by reducing cellular adenylate cyclase level.

## Discussion

Finasteride is used to treat alopecia and benign prostatic hyperplasia, owing to its ability to inhibit 5α-reductase and DHT formation in the prostate. Weinstein et al. reported that men with red hair have reduced rate of prostate cancer (Weinstein et al. 2013). Another study revealed that α-MSH and MC1R protected cell damaged by UV radiation through interaction with PTEN, thereby activating PI3K and AKT signaling (Cao et al. 2013). MC1R activity can be regulated by numerous factors, and enhanced MC1R activation can increase cAMP, PI3K and AKT signaling, thereby promoting uncontrolled cell proliferation. Based on previous reports, α-MSH and MC1R are associated with both melanogenesis and melanoma (Stanisz et al. 2011; Nasti and Timares 2014). Therefore, we evaluated the effect of finasteride on melanogenesis because it is well known that finasteride treats alopecia by inhibiting 5α-reductase.

Finasteride dose dependently inhibited melanin biosynthesis (Fig. 1a) without inducing cell death in melan-a cells (Fig 1b). Additionally, the anti-melanogenic effects of finasteride in other cell lines, specifically B16F10 melanoma cells was also measured. In B16F10 cells, melanin biosynthesis was significantly inhibited by 100 µM finasteride (Fig. 1c). In addition, treatment with 100 µM finasteride did not induced cell death nor did it change the morphology of B16F10 cells (Fig. 1d). As shown in Fig. 1c, treatment with 100 µM finasteride significantly decreased melanin content compared to cells treated with 100 nM α-MSH. These results indicated that finasteride has hypo-pigmenting effects without cytotoxicity in melanocytes and melanoma cells.

Many hypo-pigmentation agents regulate tyrosinase activity. Although melanin biosynthesis can be affected by many factors, tyrosinase is the most potent rate-limiting enzyme (Halaban et al. 1983). Further, melanin synthesis is very sensitive to stimuli or changes in the cell that affect tyrosinase (Newton et al. 2007). Therefore, tyrosinase is a very important target because it may affect other melanogenic enzymes, including TRP-1 and TRP-2 (Tsukamoto et al. 1992). To evaluate the effect of finasteride on tyrosinase, a cell free assay and an in situ assay were performed using l-DOPA as a substrate. In situ tyrosinase activity was measured after 72 h finasteride treatment and incubation with l-DOPA. The color of 10 µM finasteride treated melan-a cells was lighter than that of untreated cells (Fig. 2a, b). To evluate the effects of finasteride on human melanocytes, NHEM cells were used. Samples were treated with finasteride for 72 h and melanin synthesis was evaluated by capturing pictures. Compared to untreated NHEM cells, treatment with 10 µM finasteride decreased melanin content (Fig. 2f, g). Further, B16F10 murine melanoma cells were also evaluated. B16F10 cells were treated with finasteride and α-MSH (Yang et al. 2011). Figure 2c shows untreated B16F10 cells and Fig. 2d shows the increase in melanin induced by 100 nM α-MSH. Interestingly, treatment with 100 µM finasteride significantly reduced the melanin content to the same level as that observed in the untreated cells (Fig. 2e). Therefore, finasteride inhibited tyrosinase in both melanocytes and melanoma cells. Moreover, after treatment with 100 µM finasteride in melanoma cells, no toxicity was observed and a lower concentration of finasteride was required to observe effects in melanocytes compared to that required in melanoma cells.

Mushroom tyrosinase activity was evaluated in the presence of various concentrations of finasteride. Compared to control, tyrosinase activity dose-dependently decreased in the presence of finasteride (Fig. 2h). Surprisingly, treatment with 100 µM finasteride reduced tyrosinase activity by 20% over that of control. This efficacy was similar to that of kojic acid, a well-known tyrosinase inhibitor. In addition, tyrosinase activity in melan-a cell lysates was estimated using l-DOPA. Treatment with increasing doses of finasteride inhibited tyrosinase activity in the lysates compared to that in control (Fig. 2i). Interestingly, finasteride inhibited melan-a tyrosinase more effectively than mushroom tyrosinase. These data suggested that regulation of tyrosinase activity by finasteride was not due to differences in chemical structure. In conclusion, we propose that finasteride is a potential inhibitor of tyrosinase activity, thereby inhibiting melanin biosynthesis.

To clarify the mechanism regulating the inhibition of melanin synthesis by finasteride, we performed western blot analysis of melanogenic enzymes (Fig. 3). Among the enzymes evaluated, expression levels of tyrosinase, TRP-1 and MITF, were significantly reduced in finasteride-treated cells. In particular, treatment with a high dose of finasteride inhibited the protein expression of melanogenesis-related enzymes. As the expression of MITF decreased, we investigated the relationship between MITF, α-MSH and MC1R and an enzyme called adenylate cyclase. MC1R gene is the primary regulator of human skin pigmentation. α-MSH acts as an agonist of MC1R (Kennedy et al. 2001; Garcia-Borron et al. 2005). The increase in melanin synthesis by α-MSH through the cAMP/PKA pathway might increase MC1R gene expression, and cause changes in MITF, tyrosinase, TRP-1, and TRP-2 (Dessinioti et al. 2011). In the present study, finasteride treatment inhibited the α-MSH stimulated melanogenesis in B16F10 melanoma cells. Finasteride decreased the expression of adenylate cyclase in melan-a cells, and also inhibited adenylate cyclase expression in the α-MSH treated B16F10 cells. This indicates that finasteride controlled melanin synthesis by regulating adenylate cyclase concentration. Recently it was reported that MC1R upregulation or activation of cAMP increase cell proliferation in melanoma cells. Thus, the increased MC1R expression at 100 µM finasteride in B16F10 (Fig. 3) might be due to increased cell proliferation at 100 µM finasteride in B16F10 (Lyons et al. 2013). Chen et al. reported that the topical administration of finasteride may cause local inhibition of sebaceous gland growth in both the costovertebral organs and ears. The topical application of finasteride may also have significant systemic effects (Chen et al. 1995). Because we are concerned about the safety of finasteride, we will evaluate dose response and toxicity in later studies. Further, our results may have interesting applications in the field of cancer because this mechanism is related to carcinoma and can promote melanoma cell proliferation.

In conclusion, our study demonstrated that finasteride inhibited melanogenesis in melanocytes and melanoma cells through the down-regulation of tyrosinase, TRP-1, MITF and adenylate cyclase expression. To confirm the in vitro effects of finasteride on melanogenesis, further in vivo animal experiments are required. The present study suggested that finasteride was not only a potential whitening agent, but may also prevent skin cancer, particularly in men.
